# Electrical stimulation mitigates muscle degradation shift in gene expressions during 12-h mechanical ventilation

**DOI:** 10.1038/s41598-023-47093-w

**Published:** 2023-11-17

**Authors:** Hideki Nakai, Yutaka Hirata, Hidemasa Furue, Yoshitaka Oku

**Affiliations:** 1 Physiome, Department of Physiology, Hyogo Medical University, 1-1 Mukogawa-cho, Nishinomiya, Hyogo 663-8501 Japan; 2 Department of Rehabilitation, Hyogo Prefectural Nishinomiya Hospital, 13-9, Rokutanji, Nishinomiya, Hyogo 662-0918 Japan; 3 Department of Neurophysiology, Hyogo Medical University, 1-1 Mukogawa-cho, Nishinomiya, Hyogo 663-8501 Japan

**Keywords:** Respiration, Respiratory signs and symptoms

## Abstract

Ventilator-induced diaphragm dysfunction (VIDD), a dysfunction of the diaphragm muscle caused by prolonged mechanical ventilation (MV), is an important factor that hinders successful weaning from ventilation. We evaluated the effects of electrical stimulation of the diaphragm muscle (pulsed current with off-time intervals) on genetic changes during 12 h of MV (E-V12). Rats were divided into four groups: control, 12-h MV, sham operation, and E-V12 groups. Transcriptome analysis using an RNA microarray revealed that 12-h MV caused upregulation of genes promoting muscle atrophy and downregulation of genes facilitating muscle synthesis, suggesting that 12-h MV is a reasonable method for establishing a VIDD rat model. Of the genes upregulated by 12-h MV, 18 genes were not affected by the sham operation but were downregulated by E-V12. These included genes related to catabolic processes, inflammatory cytokines, and skeletal muscle homeostasis. Of the genes downregulated by 12-h MV, 6 genes were not affected by the sham operation but were upregulated by E-V12. These included genes related to oxygen transport and mitochondrial respiration. These results suggested that 12-h MV shifted gene expression in the diaphragm muscle toward muscle degradation and that electrical stimulation counteracted this shift by suppressing catabolic processes and increasing mitochondrial respiration.

## Introduction

Ventilator-induced diaphragmatic dysfunction (VIDD) is a dysfunction of the diaphragm that is primarily caused by atrophy of the diaphragm muscle and reduced muscle contraction due to prolonged mechanical ventilation (MV)^1^. It is frequently seen in critically ill patients requiring ventilatory management in the intensive care unit (ICU). VIDD is considered an important factor hindering successful ventilator weaning; failure of ventilator weaning leads to prolonged hospitalization and increased mortality^2^.

The diaphragm muscle weakness in VIDD must be distinguished from that caused by ICU-acquired weakness and disuse syndromes. Peripheral muscle weakness caused by ICU-acquired weakness is characterized by the presence of conduction abnormalities on electrophysiological testing^3,4^. Progressive muscle weakness in VIDD is also critically different from disuse syndrome, as systemic inflammation in VIDD affects the protein, cellular, and genetic levels^1,3,5^. The decreases in respiratory airway pressure (AP) and diaphragm mobility during inspiration produced by VIDD are thought to result in decreased spontaneous breathing capacity, leading to prolonged ventilatory management^3,6^.

Various treatment strategies for VIDD have previously been investigated in basic research and clinical settings. In basic research, activation of heat shock proteins (HSPs) by hyperthermia^7,8^, phrenic nerve stimulation^9,10^, and administration of drugs that inhibit the reactive oxygen species (ROS) signaling pathway^11–14^ have shown some efficacy. In clinical practice, the promotion of spontaneous breathing through the use of forced ventilation^15–17^, respiratory muscle training^18^, and diaphragmatic pacing^19^ has been addressed. However, no established treatment strategy exists or is undergoing further validation.

Phrenic nerve stimulation is known to improve mitochondrial oxygen utilization at the cellular level^20^ and promote protein synthesis through activation of peroxisome proliferator-activated receptor-gamma coactivator-1 alpha (PGC-1α) and other transcription factors, which in turn inhibits muscle oxidative metabolism by inflammatory cytokines^21,22^. However, these effects have not been studied in the context of VIDD, and it is necessary to verify whether this method is effective in preventing VIDD. Therefore, we created a VIDD rat model with reduced diaphragm movement due to prolonged mechanical ventilatory management; conducted a pilot study to evaluate whether direct electrical stimulation of the diaphragm muscle (ES) can counteract VIDD by observing genetic changes after prolonged MV, sham operation, and ES; and analyzed the differences in gene expression among the treatment groups.

## Methods

### Animals

The experimental protocol was approved by the animal care and use committee of Hyogo Medical University (No. 23-032A). All procedures performed on animals were in accordance with the regulations for animal experimentation of the Hyogo Medical University; with the Guidelines for Proper Conduct of Animal Experiments, Science Council of Japan; and with the ARRIVE guidelines. Adult male Wistar rats that were 14–16 weeks old and weighed 300–350 g were used in this study. The rats were purchased from Japan SLC Inc. (Shizuoka, Japan) and subsequently raised at the Disease Model Research Center under specific pathogen-free conditions to ensure their microbiological quality for the purpose of pathological model studies.

### Experimental design

Sixteen rats were randomly assigned to four groups (n = 4/group). The first group included nonventilated animals as the control (CON) group, in which the diaphragm was harvested immediately after administration of anesthesia. The second group was the ventilatory management (V12) group, in which tracheostomy was performed under anesthesia and MV was maintained for 12 h. The third group was the sham operation (S-V12) group, in which tracheostomy and midline abdominal incision were performed under anesthesia, electrodes were inserted into the diaphragm, the abdomen was closed with the lead wire remaining, and MV was managed for 12 h but ES was not applied. The fourth group was the ventilatory management and ES (E-V12) group, in which tracheostomy and midline abdominal incision were performed under anesthesia, electrodes were inserted into the diaphragm, the abdomen was closed with the lead wire remaining, continuous ES was performed, and MV was managed for 12 h while monitoring diaphragmatic contraction.

### Animal preparation

Anesthesia was first induced using isoflurane (4.0–5.0%) and then maintained with intraperitoneal urethane injection (1.2–1.5 g/kg, i.p.). If withdrawal reflexes in response to a noxious stimulus (e.g. ear pinch) were observed during the experiment, then a supplemental dose of urethane was administered intraperitoneally (0.16 g/kg) to maintain areflexia. Atropine (80–90 µg/kg) was injected intramuscularly to suppress airway secretions. Tracheostomy and intubation were performed to manage stable MV. Rats were placed on a Harvard rodent ventilator model 683 (Harvard Apparatus, Holliston, MA, USA) and ventilated for 12 h with a tidal volume of 7 ml/kg, a respiratory rate of 110–120 cycles/min, and a minute volume of 770–840 ml/kg/min, without a positive end-expiratory pressure. These ventilator settings were maintained throughout the experiment. During preliminary experiments, it was confirmed that PaO_2_ in the sham operation group and ES group tended to decrease more clearly than in the control group due to laparotomy. Therefore, supplemental oxygen was administered in the sham operation group and ES group. The oxygen flow rate was adjusted so that PaO_2_ at the start of MV did not exceed 200 mmHg to minimize the adverse effect of reactive oxygen species.

An arterial catheter was inserted into the femoral artery, and a thermometer was inserted into the rectum via the anus to measure body temperature. Body temperature was adjusted to 35.5–36.5 °C with a heating pad. During ventilator management, AP, arterial blood pressure (ABP) and body temperature were continuously monitored to ensure suppression of spontaneous breathing and stable circulation. Additional supplemental fluids were not necessary to maintain ABP within a physiological range (ABP of 80 mmHg or higher). Arterial blood gas (ABG) values were measured using an i-STAT Analyzer at the start and end of 12-h MV. After 12 h of mechanical ventilatory management, the diaphragm was isolated, and the tendon center and rib portion were dissected. The tissue surrounding the electrode, which had been inserted into the diaphragm, was removed to prepare a specimen for microarray analysis. At the end of the experiment, the animals were euthanized by whole-heart blood extraction.

### Electrical stimulation

In the present study, ES was administered to rats using an electrical stimulator (SEN-3301, Nihon Kohden, Japan). Square-wave pulse trains (200 Hz) consisting of 10 pulses with a pulse duration of 200 μs were given every 500 ms (duty cycle: 10%, on-time: 50 ms, off-time: 450 ms) to avoid muscle fatigue. Stimulation was given at an intensity of 80–100 μA using an isolator (SS-202 J, Nihon Kohden, Japan). Silver electrodes were inserted into the rib portions on the left and right sides of the diaphragm using hook electrodes, visually confirming the insertion sites from the midline abdominal incision. In the E-V12 group, ES was administered throughout the 12-h period of mechanical ventilatory management.

### Total RNA extraction from the diaphragm

The muscle tissue of the whole diaphragm isolated from rats in each group was minced using fine (iris) scissors in phosphate-buffered saline on ice. The suspension was dispensed to 100 μg of muscle tissue per microtube. After centrifugation of each tube at 11,000×*g* for 5 min at 4 °C, the supernatant was removed and stored in liquid nitrogen until use for ribonucleic acid (RNA) extraction. The minced muscle tissue was resuspended in 500 μl of RNA-Save (Biological Industries, Israel) and kept at 4 °C for 2–4 h for permeabilization. Each tube was then centrifuged at 11,000×*g* for 10 min at 4 °C, and the supernatant was removed. Total RNA was isolated from the minced muscle tissue using a NucleoSpin RNA Kit (Takara, Japan) according to the manufacturer’s instructions. The quality of the purified RNA sample in each group for microarray was evaluated with an Agilent 2100 Bioanalyzer and Agilent RNA 6000 Nano Kit (Agilent Technologies, Inc. USA). RNA samples with an RNA integrity number (RIN) > 8.0 and an A260/A280 of approximately 2.0 were used for gene expression analysis.

### Transcriptome analysis

From 250 ng of total RNA in each group, fragmented and biotin-labeled complementary deoxyribonucleic acid (cDNA) samples were synthesized using a Gene Chip WT PLUS Reagent Kit according to the protocol provided with the kit. Gene Chip arrays (Rat Clariom S) were hybridized with biotin-labeled cDNA samples at 45 °C for 16 h (60 rpm) using a Gene Chip Hybridization Oven 645. The arrays were washed and stained with a Gene Chip Fluidics Station 450 and scanned on an Affymetrix Gene Chip Scanner 3000 7G using Command Console Software. The signal values of the Gene Chip array were normalized based on the total intensity of the array using the SST-RMA algorithm implemented in Transcriptome Analysis Console (TAC) v4.0 software (Thermo Fisher Scientific, MA, USA). The raw data and the full data set are registered in the NCBI Gene Expression Omnibus with the accession number GSE244259. Additionally, quality control (QC) for each sample was performed using QC metrics in TAC v4.0. Differentially expressed genes were identified as those with a fold-change ≥ 2.0 and a p value < 0.05 using a parametric t test. We performed multivariate analysis of variance (MANOVA) using R version 4.2.3 to assess whether the CON, V12, S-V12, and E-V12 groups were significantly clustered in the microarray's three-dimensional principal component analysis (PCA) plot data that were analyzed using TAC software.

### Functional enrichment analysis

To determine the statistically significant enrichment of functions in multigene lists (including both upregulated and downregulated gene sets), we used the meta-analysis mode of Metascape (http://metascape.org, accessed on 9 June 2023). The enrichment analysis was performed using the following ontology sources: the gene ontology (GO) Biological Process database and the Kyoto Encyclopedia of Genes and Genomes (KEGG). Statistical significance was assessed using accumulative hypergeometric p values, and terms with a p value < 0.01 were depicted in a heatmap, color-coded by − log10(p value). We set a threshold of 0.3 for the kappa score and considered similarity scores > 0.3. The heatmap visualization facilitated the hierarchical clustering of statistically significant terms into GO terms, thereby highlighting commonly enriched (overlapping) and selectively enriched clusters and pathways across multiple gene lists. This comprehensive approach offered by Metascape enabled a thorough assessment of the molecular features associated with each biological process.

### Statistical analysis

All statistical analyses were conducted using R version 4.2.3. Data are indicated by means and standard deviations. Statistically significant differences (p < 0.05, Fig. 4) among the four groups (CON, V12, S-V12, E-V12) from the PCA plot data of the microarray were confirmed using MANOVA with R version 4.2.3, with each group in the animal experiment having a sample size of 4. Since this sample size also satisfied the minimal requirement of data points (n = 3) to calculate Tukey’s biweight average, we proceeded to conduct microarray analysis using TAC.

Rat body weight, ABP, and ABG were compared using the Kruskal‒Wallis test, followed by post hoc pairwise comparisons using the Mann‒Whitney U test. The p values were adjusted using the Bonferroni correction to control the inflated type I error rate resulting from multiple comparisons. The Wilcoxon signed rank test was used for comparisons of ABG parameter values between the start and end of experiments. P values of < 0.05 were considered to indicate statistical significance.

## Results

### Overall characterization of changes in ABG parameters and gene expression during V12, S-V12, and E-V12

**Figure 1:**
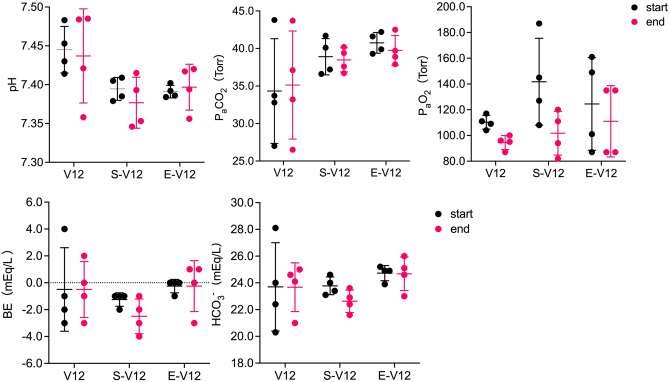
ABG parameter values at the start and end of 12-h MV in the V12, S-V12, and E-V12 groups. ABG parameter values at the start (black bar) and end (pink bar) of 12-h MV in the V12, S-V12, and E-V12 groups are shown. Pairwise comparisons among V12, S-V12, and E-V12 conducted using the Mann‒Whitney *U* test with the Bonferroni correction did not show statistically significant differences for all ABG parameters.

The ABG parameter values at the start and end of 12-h MV during V12, S-V12, and E-V12 are shown in Fig. 1. Although significant differences were observed in the Kruskal‒Wallis test for pH at the start, no significant differences were observed for any ABG parameters at either the start or end of 12-h MV in pairwise comparisons among V12, S-V12, and E-V12 using the Mann‒Whitney *U* test. Comparisons of ABG parameter values between the start and end of 12-h MV also showed no significant differences in the V12, S-V12, and E-V12 groups.

**Figure 2:**
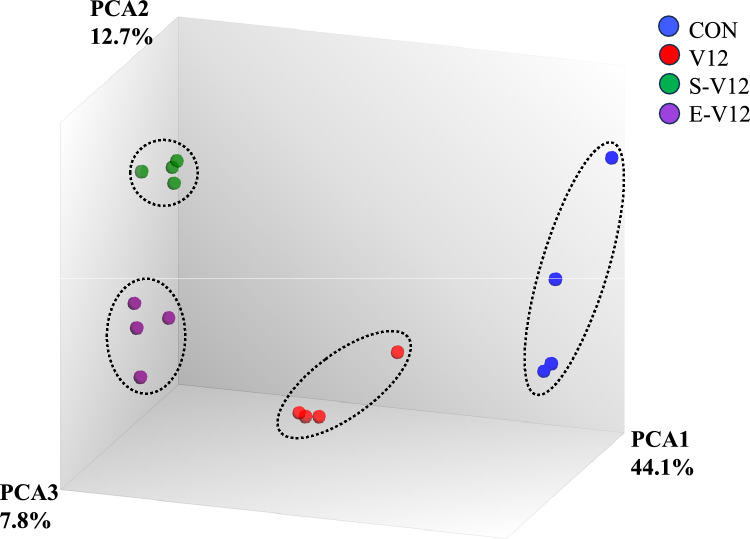
PCA plot of microarray expression data. The figure represents the three principal components (PC1, PC2 and PC3) of the microarray analysis data. Each circle indicates the normalized gene expression of one microarray sample, and the color of the circle indicates the treatment condition. CON, V12, S-V12, and E-V12 samples (n = 4) were significantly clustered based on their respective groups by MANOVA.

**Figure 3:**
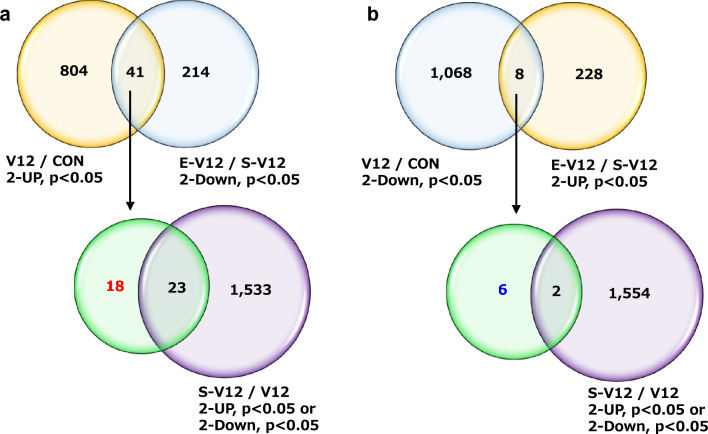
Venn diagram of genes altered by ES among the differentially expressed genes in the diaphragm in the VIDD rat model. (**a**) Out of the 845 upregulated genes in the V12/CON comparison, 41 genes that overlapped with the 255 downregulated genes in E-V12/S-V12 were downregulated by ES. Excluding the overlap with 1556 differentially expressed genes affected by the laparotomy procedure of ES resulted in 18 genes. (**b**) Out of the 1076 downregulated genes in V12/CON, 8 genes that overlapped with the 236 upregulated genes in E-V12/S-V12 were upregulated by ES. Excluding the overlap with 1556 differentially expressed genes affected by the laparotomy procedure of ES resulted in 6 genes.

As shown in Fig. 2, the CON, V12, S-V12, and E-V12 groups were significantly clustered in the 3-dimensional PCA plot of the microarray data (Pillai’s trace = 2.4838, approx F = 19.247, df = 3, 36, p = 3.001e-11). There were 1921 differentially expressed genes (845 upregulated, 1076 downregulated) in the V12 group (V12/CON), 1556 differentially expressed genes (925 upregulated, 631 downregulated) in the S-V12 group (S-V12/V12), and 491 differentially expressed genes (236 upregulated, 255 downregulated) in the ES group (E-V12/S-V12), as shown in the Venn diagrams (Fig. 3a and b).

### Functional annotation and enrichment analysis

**Figure 4:**
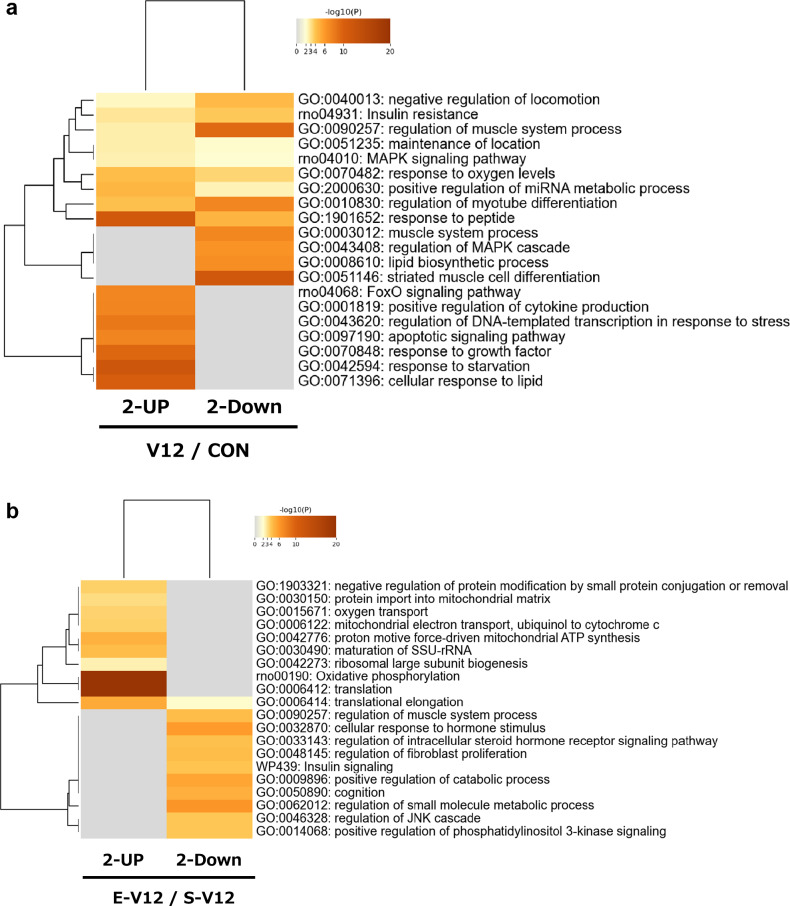

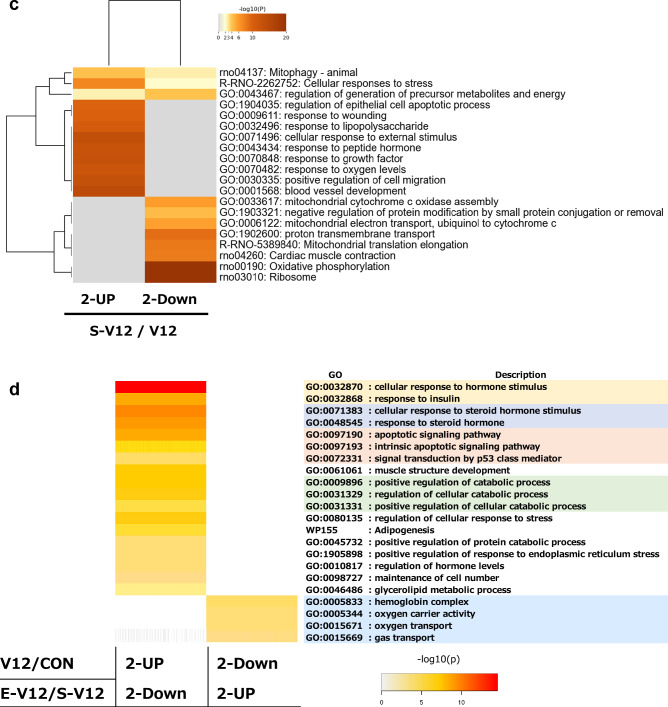
Functional enrichment analysis for genes altered by ES among differentially expressed genes in the diaphragm in the VIDD rat model. Pathway and process enrichment analyses were performed using Metascape (http://metascape.org, accessed on 9 June 2023) for (**a**) the 1921 genes (845 upregulated and 1076 downregulated genes) in V12/CON, (**b**) the 491 genes (236 upregulated and 255 downregulated genes) in E-V12/S-V12, (**c**) the 1556 genes (925 upregulated and 631 downregulated genes) in S-V12/V12, and (**d**) the 18 and 6 genes as illustrated in the Venn diagrams in Fig. 3a and b. The heatmaps display the results of enrichment analysis of all statistically enriched ontology terms (GO biological processes, KEGG pathways, Reactome Gene Sets, WikiPathways), with the color representing the − log10(p value). The thresholds used were a 0.3 kappa score and a similarity score greater than 0.3 (**a**–**c**). Darker shades of orange indicate greater significance for the term, while a gray color indicates a lack of significance. The dendrograms show the closeness of the enriched term clusters, with one per row. The grouped colors represent the clustering of enriched GO terms, and the heatmap visualizes the GO results of Metascape using heatmapper (http://www.heatmapper.ca/expression/) (**d**).

We utilized Metascape to conduct annotation and enrichment analyses on multiple gene sets derived from the diaphragm in the VIDD rat model. As shown in the heatmap representations (Fig. 4a), we observed significant functional overlap, with 9 out of the top 20 enriched terms corresponding to the same biological processes present in the 1921 genes (845 upregulated and 1076 downregulated genes) in the V12/CON comparison. Interestingly, for the downregulated genes (1076 genes), we identified five selectively enriched pathways, including "muscle system process" and "striated muscle cell differentiation", both of which are associated with muscle function. In contrast, the upregulated genes (845 genes) did not exhibit significant enrichment in muscle-related GO terms. Instead, we identified seven selectively enriched clusters and pathways, including the "forkhead box protein O1 (FoxO1) signaling pathway", "positive regulation of cytokine production", and the “apoptotic signaling pathway”. Table 1a shows a list of genes that were duplicated among the significantly enriched GO terms in the V12/CON comparison and the fold changes corresponding to the list of overlapping genes in the microarray.

**Table 1:** List of the significantly enriched gene ontology terms corresponding to differences in gene expression.

(a) Regulated genes in V12/CON	GO term	GO term	Gene symbol	
2-UP	rno04068	GO:0071396	Fbxo32, Foxo1, Foxo3, Mapk14, Pik3r1, Sgk1	
	rno04068	GO:0070848	Crebbp, Foxo3, Mapk14, Pik3r1, Sirt1, Smad3, Stat3	
	rno04068	GO:0090257	Fbxo32, Foxo1, Foxo3, Sirt1, Smad3	
	GO:0070848	GO:0090257	Errfi1, Foxo3, Mstn, Myog, Rock2, Runx1, Sirt1, Smad3	
	GO:0071396	GO:0090257	Errfi1, Fbxo32, Foxo1, Foxo3, Mstn, Myog, Pde4b, Pde4d, Rock2, Tnfrsf1a, Trim63, Zc3h12a	
	GO:0042594	GO:0001819	Atf4, Cebpb, Egr1, Rela	
2-Down	GO:0003012	GO:0010830	Cav3, Csrp3, Dmpk, Lmod3, Myod1, Trim72	
	GO:0003012	GO:0090257	Adra1a, Adrb2, Ank2, Atp1b1, Casq2, Kcnj2, Kcnma1, Mef2c, Ncf1, Nr3c1, Pkp2, Ppargc1a, Prkg1, Rgs2	

Regulated genes in V12/CON	Gene symbol	Fold change	p-val	Description
2-UP	Errfi1	75.47	1.0.E-15	ERBB receptor feedback inhibitor 1
	Runx1	55.37	3.5.E-14	Runt-related transcription factor 1
	Foxo1	37.80	4.4.E-14	Forkhead box O1
	Mstn	16.01	4.3.E-11	Myostatin
	Trim63	15.29	7.5.E-13	Tripartite motif containing 63, E3 ubiquitin protein ligase
	Pde4b	11.44	6.4.E-12	Phosphodiesterase 4B, cAMP specific
	Sgk1	10.34	7.6.E-08	Serum/glucocorticoid regulated kinase 1
	Mapk14	9.00	5.6.E-13	Mitogen activated protein kinase 14
	Egr1	8.85	5.0.E-04	Early growth response 1
	Myog	8.73	1.9.E-11	Myogenin
	Stat3	8.28	1.3.E-11	Signal transducer and activator of transcription 3 (acute-phase response factor)
	Pde4d	6.47	3.1.E-08	Phosphodiesterase 4D, cAMP-specific
	Atf4	4.25	1.3.E-10	Activating transcription factor 4
	Sirt1	4.09	9.9.E-10	Sirtuin 1 (Silent mating type information regulation 2, homolog) 1
	Fbxo32	3.62	2.8.E-08	F-box protein 32
	Smad3	3.43	4.1.E-08	SMAD family member 3
	Cebpb	3.25	1.1.E-06	CCAAT/enhancer binding protein (C/EBP), beta
	Rela	3.15	1.4.E-07	v-rel avian reticuloendotheliosis viral oncogene homolog A
	Tnfrsf1a	3.15	8.5.E-08	Tumor necrosis factor receptor superfamily, member 1a
	Zc3h12a	3.13	1.4.E-06	Zinc finger CCCH type containing 12A
	Rock2	2.85	2.5.E-08	Rho-associated coiled-coil containing protein kinase 2
	Pik3r1	2.61	1.2.E-05	Phosphoinositide-3-kinase, regulatory subunit 1 (alpha)
	Foxo3	2.41	1.3.E-04	Forkhead box O3
	Crebbp	2.28	4.0.E-05	CREB binding protein
	Tp53	2.09	8.7.E-06	Tumor protein p53
2-Down	Rgs2	− 2.13	1.9.E-04	Regulator of G-protein signaling 2
	Mef2c	− 2.15	1.4.E-04	Myocyte enhancer factor 2C
	Pkp2	− 2.21	9.2.E-06	Plakophilin 2
	Kcnma1	− 2.25	6.6.E-05	Potassium channel, calcium activated large conductance subfamily M alpha, member 1
	Trim72	− 2.30	9.9.E-06	Tripartite motif containing 72, E3 ubiquitin protein ligase
	Atp1b1	− 2.32	8.8.E-06	ATPase, Na + /K + transporting, beta 1 polypeptide
	Ank2	− 2.34	1.9.E-05	Ankyrin 2
	Dmpk	− 2.47	3.0.E-05	Dystrophia myotonica-protein kinase
	Cav3	− 2.57	4.2.E-06	Caveolin 3
	Myod1	− 2.57	7.6.E-06	Myogenic differentiation 1
	Prkg1	− 2.88	4.9.E-08	Protein kinase, cGMP-dependent, type 1
	Casq2	− 2.96	4.5.E-05	Calsequestrin 2 (cardiac muscle)
	Adra1a	− 3.48	2.3.E-08	Adrenoceptor alpha 1A
	Lmod3	− 4.50	6.8.E-08	Leiomodin 3 (fetal)
	Adrb2	− 4.79	5.4.E-08	Adrenoceptor beta 2, surface
	Kcnj2	− 4.97	5.1.E-08	Potassium channel, inwardly rectifying subfamily J, member 2
	Nr3c1	− 6.56	7.4.E-12	Nuclear receptor subfamily 3, group C, member 1
	Ncf1	− 9.22	2.8.E-11	Neutrophil cytosolic factor 1
	Ppargc1a	− 37.13	1.0.E-09	Peroxisome proliferator-activated receptor gamma, coactivator 1 alpha (PGC1-α)

(b) Regulated genes in E-V12/S-V12	GO term		Gene symbol	
2-UP	GO:0006122	rno00190	Uqcrh, Uqcr10, Uqcrb, Atp5f1e	
	GO:0042776	rno00190	Atp5f1e, Atp5mg, Atp5pf, Atp5pd	
2-Dwon	GO:0009896	GO:0062012	Sox9, Cpt1a, Ldlr, Gpd1, Pink1, Prkn, Mlxipl, Git1	
	GO:0032870	GO:0062012	Ppara, Wdtc1, Pdk2, Ppard, Nr1d1	

Regulated genes in E-V12/S-V12	Gene symbol	Fold change	p-val	Description
2-UP	Atp5f1e	3.34	6.1.E-04	ATP synthase F1 subunit epsilon
	Uqcrb	2.44	2.2.E-05	Ubiquinol-cytochrome c reductase binding protein
	Atp5pd	2.20	5.0.E-04	ATP synthase peripheral stalk subunit d
	Atp5mg	2.13	4.3.E-03	ATP synthase membrane subunit g
	Atp5pf	2.07	1.2.E-03	ATP synthase peripheral stalk subunit F6
	Uqcrh	2.05	2.5.E-03	Ubiquinol-cytochrome c reductase hinge protein
	Uqcr10	2.03	3.0.E-04	Ubiquinol-cytochrome c reductase, complex III subunit X
2-Down	Ppara	− 2.01	1.6.E-02	Peroxisome proliferator activated receptor alpha
	Git1	− 2.06	1.3.E-02	G protein-coupled receptor kinase interacting ArfGAP 1
	Cpt1a	− 2.11	6.3.E-03	Carnitine palmitoyltransferase 1a, liver
	Pdk2	− 2.14	2.1.E-03	Pyruvate dehydrogenase kinase, isozyme 2
	Gpd1	− 2.18	1.2.E-03	Glycerol-3-phosphate dehydrogenase 1 (soluble)
	Pink1	− 2.22	8.4.E-04	PTEN induced putative kinase 1
	Ppard	− 2.22	7.5.E-04	Peroxisome proliferator-activated receptor delta
	Mlxipl	− 2.25	5.4.E-04	MLX interacting protein-like
	Wdtc1	− 2.29	2.4.E-04	WD and tetratricopeptide repeats 1
	Ldlr	− 2.44	1.4.E-04	Low density lipoprotein receptor
	Nr1d1	− 2.60	5.4.E-05	Nuclear receptor subfamily 1, group D, member 1
	Prkn	− 2.72	3.4.E-05	Parkin RBR E3 ubiquitin protein ligase
	Sox9	− 3.07	2.1.E-05	SRY (sex determining region Y)-box 9

(c) Regulated genes in S-V12/V12	GO term			Gene symbol
2-UP	GO:0071496	GO:0070482		Ankrd1, Cdkn1a, Dnm1l, Egr1, Eng, Fos, Foxo3, Hif1a, Hmox1, Insr, Jun, Lcn2, Map3k7, Nfe2l2, Ppara, Ppard, Ppp1r15a, Prkaa1, Serpine1, Sfrp1, Slc2a1, Slc7a5, Srf, Tnfrsf1a
	GO:0032496	GO:0043434		Bcl2l1, Ccl2, Ednrb, Fn1, Fos, Hif1a, Il6r, Jun, Jund, Mapk14, Nr4a1, Serpina3n, Serpine1, Socs3, Xbp1
2-Down	GO:1902600	rno04260	rno00190	Uqcr10, Uqcrh, Uqcrb, Cox5a, Cox5b, Cox8b, Cox6a2, Cox6c

Regulated genes in S-V12/V12	Gene symbol	Fold change	p-val	Description
2-UP	Socs3	41.78	2.8.E-10	Suppressor of cytokine signaling 3
	Egr1	14.00	2.0.E-05	Early growth response 1
	Slc7a5	13.05	4.1.E-10	Solute carrier family 7, member 5
	Serpine1	11.83	1.8.E-10	Serpin peptidase inhibitor, clade e, member 1
	Slc2a1	7.82	2.3.E-09	Solute carrier family 2 (facilitated glucose transporter), member 1
	Serpina3n	7.56	2.0.E-05	Serine (or cysteine) peptidase inhibitor, clade a, member 3n
	Nr4a1	4.81	2.2.E-02	Nuclear receptor subfamily 4, group a, member 1
	Ppard	4.71	4.5.E-07	Peroxisome proliferator-activated receptor delta
	Ankrd1	4.65	7.5.E-04	Ankyrin repeat domain 1
	Fos	4.34	1.4.E-06	FBJ osteosarcoma oncogene
	Fn1	4.23	9.8.E-07	Fibronectin 1
	Bcl2l1	4.21	9.8.E-09	Bcl2-like 1
	Il6r	4.17	1.2.E-08	Interleukin 6 receptor
	Ppp1r15a	4.01	2.2.E-07	Protein phosphatase 1, regulatory subunit 15A
	Prkaa1	3.89	2.8.E-09	Protein kinase, AMP-activated, alpha 1 catalytic subunit
	Tnfrsf1a	3.66	2.1.E-06	Tumor necrosis factor receptor superfamily, member 1a
	Sfrp1	3.53	1.0.E-05	Secreted frizzled-related protein 1
	Ccl2	3.50	2.4.E-03	Chemokine (C–C motif) ligand 2
	Hmox1	3.26	6.3.E-04	Heme oxygenase 1
	Ednrb	3.04	6.8.E-06	Endothelin receptor type B
	Cdkn1a	2.78	1.4.E-04	Cyclin-dependent kinase inhibitor 1A
	Jun	2.68	5.5.E-04	Jun proto-oncogene
	Foxo3	2.48	1.2.E-04	Forkhead box O3
	Insr	2.44	2.2.E-05	Insulin receptor
	Mapk14	2.40	1.7.E-07	Mitogen activated protein kinase 14
	Alpl	2.30	2.4.E-04	Alkaline phosphatase, liver/bone/kidney
	Srf	2.27	1.8.E-03	Serum response factor
	Eng	2.26	3.5.E-04	Endoglin
	Jund	2.26	1.3.E-05	Jun D proto-oncogene
	Map3k7	2.21	4.4.E-05	Mitogen activated protein kinase kinase kinase 7
	Dnm1l	2.16	1.4.E-06	Dynamin 1-like
	Nfe2l2	2.14	2.1.E-05	Nuclear factor, erythroid 2-like 2
	Hif1a	2.14	4.4.E-05	Hypoxia-inducible factor 1, alpha subunit
	Lcn2	2.11	1.6.E-04	Lipocalin 2
	Xbp1	2.09	1.1.E-04	X-box binding protein 1
	Ppara	2.08	4.6.E-03	Peroxisome proliferator activated receptor alpha
2-Down	Cox5a	− 2.02	9.0.E-06	Cytochrome c oxidase subunit 5A
	Uqcrh	− 2.03	6.7.E-04	Ubiquinol-cytochrome c reductase hinge protein
	Cox5b	− 2.13	4.7.E-06	Cytochrome c oxidase subunit 5B
	Cox6c	− 2.15	1.6.E-05	Cytochrome c oxidase subunit 6C
	Cox6a2	− 2.18	7.0.E-07	Cytochrome c oxidase subunit 6A2
	Cox8b	− 2.20	3.4.E-05	Cytochrome c oxidase, subunit viiib
	Uqcr10	− 2.51	2.0.E-06	Ubiquinol-cytochrome c reductase, complex III subunit X
	Uqcrb	− 2.79	1.4.E-06	Ubiquinol-cytochrome c reductase binding protein

Overlapping genes for two GO terms significantly enriched in V12/CON (Fig. 4a, Table 1a), E-V12/S-V12 (Fig. 4b, Table 1b), and S-V12/V12 (Fig. 4c, Table 1c). The corresponding fold change values in the microarray are shown. Significance (p < 0.05) was determined using a parametric *t* test.

In the E-V12/S-V12 comparison, selectively enriched clusters were observed for both the 236 upregulated and 255 downregulated genes (Fig. 4b). For the 236 upregulated genes, biological processes related to oxygen and mitochondria were enriched, including "protein import into mitochondrial matrix", "oxygen transport", and "mitochondrial electron transport (ubiquinol to cytochrome c)". For the 255 downregulated genes, selective enrichment was found in processes related to muscles and hormones, such as "regulation of muscle system process", "cellular response to hormone stimulus", "regulation of intracellular steroid hormone receptor signaling pathway", and "insulin signaling". Table 1b shows a list of genes that were duplicated among the significantly enriched GO terms in the E-V12/S-V12 comparison and the fold changes corresponding to the list of overlapping genes in the microarray.

In the S-V12/V12 comparison, a total of 1556 genes (925 upregulated and 631 downregulated genes) were analyzed for enrichment (Fig. 4c). For the upregulated genes and downregulated genes, there were 9 and 8 selectively enriched clusters, respectively. The 925 upregulated genes showed enrichment in clusters related to stress, tissue damage, and apoptosis, such as "cellular responses to stress", "regulation of epithelial cell apoptotic process", "response to wounding", and "cellular response to external stimulus". In contrast, the 631 downregulated genes were selectively enriched in clusters related to mitochondrial function and oxidation, including "mitochondrial cytochrome c oxidase assembly" and "mitochondrial translation elongation." Table 1c shows a list of genes that were duplicated among significantly enriched GO terms in the S-V12/V12 comparison and the fold changes corresponding to the list of overlapping genes in the microarray.

### Gene changes related to inflammatory cytokines, stress, and skeletal muscle occurred after 12-h MV, sham operation, and ES

We investigated genes associated with inflammatory cytokines, stress, and skeletal muscle according to the results of enrichment analyses. Table 2 summarizes the genes that were selected from selectively enriched clusters in the 12-h MV (V12/CON), sham operation (S-V12/V12), and ES (E-V12/S-V12) conditions, as shown in Table 1a to c. After 12 h of MV management, the expression of FoxO1 was increased by 37.8-fold, and the expression of Ppargc1a (the gene symbol of PGC1-α) was decreased by 37.1-fold. Genes associated with muscle atrophy, such as myostatin (Mstn), tripartite motif-containing 63 (Trim63), and f-box protein 32 (Fbxo32), and genes associated with inflammatory cytokines and stress, such as mitogen-activated protein kinase 14 (Mapk14), sirtuin 1 (Sirt1), and CCAAT/enhancer binding protein beta (Cebpb), were upregulated, whereas genes related to muscle synthesis, regeneration, and contraction, such as tripartite motif-containing 72 (Trim72), calsequestrin 2 (Casq2), and myogenic differentiation 1 (Myod1), were downregulated. The expression of FoxO1 and PGC1-α, which was particularly changed after 12 h of MV, was altered by no more than twofold by additional sham operation or ES (FoxO1 expression increased by 1.9-fold in the sham operation group and decreased by 1.9-fold in the ES group; PGC1-α expression decreased by 1.1-fold in the sham operation group and by 1.6-fold in the ES group). Sham operation resulted in a 7.8-fold increase in the expression of an energy metabolism-related gene, solute carrier family 2 member 1 (Slc2a1), and twofold increases in the expression of stress response genes, such as forkhead box O3 (FoxO3), Mapk14, and nuclear factor erythroid 2-related factor 2 (Nfe212). Of the 4 genes that showed twofold or greater decreases in expression under ES, the gene that showed opposite changes in expression under 12-h MV was peroxisome proliferator-activated receptor delta (Ppard). Ppard expression was increased by 1.7-fold by 12-h MV, increased by 4.7-fold by the sham operation, and decreased by 2.2-fold by ES.

**Table 2:** List of differentially expressed genes after 12-h MV, sham operation, and ES.

Regulated genes	Gene symbol	Fold change	P-val	Description
V12/CON (12-h MV)	Foxo1	37.80	4.E-14	Forkhead box O1
	Mstn	16.01	4.E-11	Myostatin
	Trim63	15.29	8.E-13	Tripartite motif containing 63, E3 ubiquitin protein ligase
	Mapk14	9.00	6.E-13	Mitogen activated protein kinase 14
	Sirt1	4.09	1.E-09	Protein Sirt1
	Fbxo32	3.62	3.E-08	F-box protein 32
	Cebpb	3.25	1.E-06	CCAAT/enhancer binding protein (C/EBP), beta
	Nfe2l2	2.89	3.E-05	Nuclear factor, erythroid 2-like 2
	Foxo3	2.41	1.E-04	Forkhead box O3
	Slc2a1	2.00	3.E-02	Solute carrier family 2, member 1
	Trim72	− 2.30	1.E-05	Tripartite motif containing 72, E3 ubiquitin protein ligase
	Myod1	− 2.57	8.E-06	Myogenic differentiation 1
	Casq2	− 2.96	5.E-05	Calsequestrin 2
	Ppargc1a	− 37.13	1.E-09	Peroxisome proliferator-activated receptor gamma, coactivator 1 alpha (PGC1-α)
E-V12/S-V12 (electric stimulation)	Ppard	− 2.22	1.E-03	Peroxisome proliferator-activated receptor delta
S-V12/V12 (sham operation)	Slc2a1	7.82	2.E-09	Solute carrier family 2, member 1
	Ppard	4.71	5.E-07	Peroxisome proliferator-activated receptor delta
	Foxo3	2.48	1.E-04	Forkhead box O3
	Mapk14	2.40	2.E-07	Mitogen activated protein kinase 14
	Nfe2l2	2.14	2.E-05	Nuclear factor, erythroid 2-like 2

Cytokine-, stress-, and muscle atrophy-related genes were selected from Table 1a to c. Related genes in the V12/CON, E-V12/S-V12, and S-V12/V12 comparisons were identified with thresholds of a fold-change ≥ 2.0 and a p value < 0.05 using a parametric *t* test.

### Genes for which ES counteracted expression changes during 12-h MV

For the ES group, laparotomy was required to insert electrodes directly into the diaphragm to ensure contraction of the diaphragm muscle. Therefore, the influence of laparotomy was accounted for to identify genes for which ES counteracted expression changes during 12-h MV.

Of the 845 genes whose expression increased by more than twofold in the 12-h MV group and the 255 genes whose expression decreased by more than twofold in the ES group, 41 genes were duplicated. The 23 genes that were upregulated or downregulated by more than twofold in the sham operation group were removed, and the remaining 18 genes were extracted as those whose expression was increased by 12-h MV but partially restored by ES (Fig. 3a). Of the 1076 genes that showed a twofold or greater decrease in expression after 12-h MV and the 236 genes that showed a twofold or greater increase in expression after ES, 8 genes were duplicates. The genes whose expression decreased by twofold or greater after the sham operation were removed, after which the number of genes whose downregulation due to 12-h MV was at least partially counteracted by ES was 6 (Fig. 3b). To summarize the Venn diagram analysis, a total of 24 of the genes that were altered by 12-h MV (18 genes upregulated by 12-h MV but downregulated by ES; 6 genes downregulated by 12-h MV but upregulated by ES) were not affected by the sham operation but were affected by ES.

For further analysis, we focused on 1100 genes (including 42 duplicates), consisting of 845 upregulated genes in V12/CON and 255 downregulated genes in E-V12/S-V12 (Fig. 3a), and 1312 genes (including 8 duplicates), consisting of 1076 downregulated genes in V12/CON and 236 upregulated genes in E-V12/S-V12 (Fig. 3b). We analyzed the commonly enriched pathways for the 1100 genes and the 1312 genes. Consequently, 18 and 6 overlapping genes whose expression changes were counteracted by ES in the VIDD rat model were found in the five identified clustering GOs (Fig. 4d). Details of the 24 genes (overlap of 18 and 6 genes) are summarized in Table 3a. For the clustered GOs, the classifications are summarized in Table 3b, with colors representing the clustering of enriched GO terms.

**Table 3:**
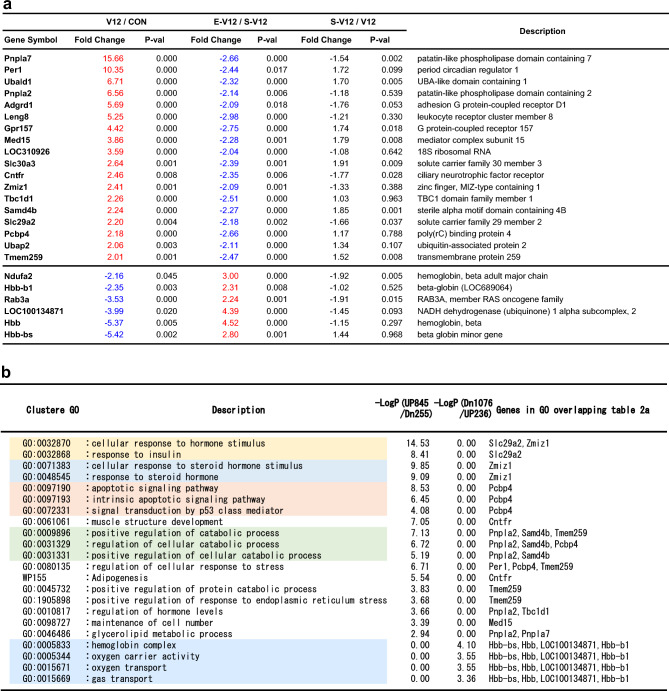
Functional enrichment analysis of genes altered by ES among differentially expressed genes in the diaphragm in the VIDD rat model.

(a) The list shown in Fig. 3a and b consists of two sets of genes: one with 18 overlapping genes, which were upregulated by 12-h MV and downregulated by ES, and the other with 6 overlapping genes, which were downregulated by 12-h MV and upregulated by ES. The two overlapping gene sets were identified with thresholds of a fold-change ≥ 2.0 and a p value < 0.05 using a parametric *t* test. (b) Regarding the functional annotation and enrichment analysis of the 1100 genes (845 upregulated genes in V12/CON and 255 downregulated genes in E-V12/S-V12) and the 1312 genes (1076 downregulated genes in V12/CON and 236 upregulated genes in E-V12/S-V12), the overlapping genes for the clustered GO terms in the heatmap are found in the gene lists presented in Table 3a.

Among the genes included in the selectively enriched GO terms, the gene associated with stress was periodic circadian clock 1 (Per1), those associated with cytokines were ciliary neurotrophic factor receptor (Cntfr) and Per1, and those associated with skeletal muscle were Cntfr and patatin-like phospholipase domain-containing 7 (Pnpla7). Other changes in gene expression were observed for genes associated with the apoptotic signaling pathway, catabolic process, hormones, and muscle. The six genes that were downregulated by 12-h MV and upregulated by ES were related to internal respiration (mitochondrial respiration and oxygen transport). The gene related to mitochondrial respiration was NADH:ubiquinone oxidoreductase subunit A2 (Ndufa2), and the genes related to oxygen transport were hemoglobin genes.

## Discussion

This is the first study to examine the effects of ES during prolonged MV using genetic analysis of the diaphragm muscle. Although there have been numerous studies related to ES-induced effects on muscle atrophy^21,23,24^, no study has analyzed the genetic changes caused by ES during prolonged MV. The main findings of the present study are that 12-h MV is reasonable for establishing a VIDD rat model and that ES favorably affects genes related to inflammatory cytokines, skeletal muscle, and internal respiration (oxygen transport and mitochondrial respiration) during prolonged MV.

Our study showed that 12 h of MV caused activation of forkhead box O (FoxO) signaling and suppression of PGC1-α. In addition, muscle atrophy genes (Mstn, Trim63, Fbxo3) and inflammatory cytokine and stress genes (Mapk14, Sirt1, Cebpb) were upregulated, while skeletal muscle regeneration and repair genes (Trim72, Casq2, Myod1) were downregulated. Previous studies have shown that 12-h MV causes diaphragmatic muscle atrophy, resulting in decreased ventilatory capacity due to reduced muscle strength and fiber loss^1,25^. MV induces significant muscle weakness and loss of muscle fibers within 12 h in rats and within 2 days in humans^1^. FoxO-family transcription factors play critical roles in the loss of muscle mass^26^. For example, transgenic mice specifically overexpressing FoxO1 in skeletal muscle have reduced skeletal muscle mass^27^. Furthermore, FoxO1 regulates the expression of myostatin and contributes to the control of muscle cell growth and differentiation^28^. When FoxO3 is activated, it induces the expression of ubiquitin ligases associated with atrophy (e.g. those known for acting as signals for proteasome-dependent degradation of target proteins), leading to a significant decrease in muscle mass. PGC-1α protects skeletal muscle from atrophy by suppressing FoxO3 action and atrophy-specific gene transcription^29^. On the other hand, FoxO1 may interact with PGC-1α to inhibit certain functions of PGC-1α, inhibiting the expression of slow-fiber genes^30^. The 12-h MV in the present study caused results compatible with those reported in previous studies, suggesting that our model is a reasonable VIDD rat model.

In our study, 12-h MV resulted in the upregulation of genes related to inflammatory cytokines and stress and the downregulation of genes controlling skeletal muscle homeostasis (regeneration and repair), in addition to an increase in muscle degradation and a decrease in muscle synthesis. These changes in gene expression may have been closely related. The mechanism of VIDD is thought to involve (1) an increase in ROS accumulation in mitochondria, which induces apoptosis via the oxidative effect on proteins, and (2) the effect of transcription factor-induced gene activity on the progression of atrophy, which leads to protein degradation in the diaphragm muscle^1^. Moreover, it has been reported that prolonged MV increases the levels of inflammatory cytokines by activating the nuclear factor-kappaB (Nf-kB) pathway in diaphragm tissue^31^. The increases in inflammatory cytokine levels promote muscle degradation and inhibit synthesis^32^.

In this study, a laparotomy was performed to apply ES, and electrodes were inserted directly into the diaphragm. Therefore, it was necessary to distinguish between ES-induced genetic changes and those caused by the operation. Sham operation increased energy metabolism gene (Slc2a1) and stress gene (FoxO3, Mapk14, Nfe212) expression. The results indicate that invasive laparotomy increases metabolism and stress and may also affect muscle structure.

We identified 18 genes that showed twofold or greater increases in expression during 12 h of ventilatory management, were unaffected by the sham operation, and showed twofold or greater decreases in expression during ES. These included genes associated with catabolic processes, inflammatory cytokines, stress, and skeletal muscle (energy metabolism). Of note, among the genes associated with three enriched GO terms related to catabolic processes, Pnpla2, Samd4b, Tmem259, and Pcbp4 were upregulated by 12-h MV and downregulated by ES. Pnpla7, the gene whose expression was most altered by 12-h MV, regulates skeletal muscle energy metabolism by inversely correlating with insulin^33^. Therefore, ES might counteract the catabolic process caused by 12-h MV.

We identified six genes that showed decreases in expression of more than twofold after 12 h of ventilatory management and increases of more than twofold after ES. These included genes associated with internal respiration (mitochondrial respiration and oxygen transport-related genes). It has been reported that ES may protect mitochondrial function during MV by contributing to skeletal muscle homeostasis^21^. Martin et al.^20^ reported that ES of the diaphragm during surgery in human subjects improved mitochondrial respiration by maintaining diaphragmatic contraction. Tanaka et al.^22^ showed that ES influenced mitochondria and suppressed the oxidative metabolism of muscles caused by inflammatory cytokines. Suppression of oxidative metabolism in muscles leads to reduced inflammation and damage, muscle recovery, maintenance, and growth^32^. The activation of Ndufa2 is influenced by the decreases in inflammatory cytokine levels caused by ES, leading to improvement in mitochondrial respiration^22,34^. Thus, ES during MV might protect mitochondrial respiration and prevent increases in inflammatory cytokine levels^21,22^. Increased inflammatory cytokine levels due to long-term MV have been reported to lead to serious respiratory complications, such as acute respiratory distress syndrome (ARDS) and ventilator-induced lung injury (VILI)^35,36^. Preventing increases in the levels of inflammatory cytokines is therefore of great clinical significance.

In the present study, ES increased the expression of the hemoglobin gene, which encodes a member of the oxygen transport system, in addition to mitochondrial respiration. Hemoglobin is very well known for its role in oxygen transport, but it also plays a role in inhibiting increases in nitric oxide levels in the alveolar epithelium^37^. The relationship among hemoglobin, the lungs, and muscle tissue remains to be clarified, and further investigation is needed to determine the pathways affected by ES.

We identified genes that were not upregulated by more than twofold by 12 h of MV but were downregulated by more than twofold by ES. Among them, Ppard is a gene that encodes a protein known as a transcription factor that plays important roles in inflammatory cytokines, stress, and muscle function. McClung et al.^11^ reported that 12 h of MV significantly reduced slow muscle fibers, a type 1 fiber closely related to mitochondrial respiration. Thus, continuous stimulation with ES may prevent type 1 fibers from undergoing atrophy. However, previous studies in humans have shown that MV for more than 18 h causes muscle atrophy in both type I and type II fibers^38^ and that ES recruits motor units in a nonselective, spatially fixed, and temporally synchronous pattern, which contributes to greater muscle fatigue than that occurring with voluntary actions^39^. This will need to be considered when applying ES in clinical settings. In this study, Ppard, whose expression was not increased by more than twofold during 12-h MV, was found to be upregulated by 4.7-fold in the sham operation group and downregulated by 2.2-fold in the ES group. Therefore, changes in Ppard gene expression might have attenuated the increases in inflammatory cytokine levels induced by the sham operation.

In the present study, upregulation of muscle atrophy genes and suppression of muscle synthesis genes were observed during 12-h MV, but neither had a magnitude greater than twofold under ES. FoxO1 showed a − 1.9-fold decreasing trend with ES, but PGC1-α showed no genetic changes antagonistic to those under 12-h MV. ES has been shown to activate the PGC1-α pathway of muscle synthesis proteins, preventing muscle protein degradation and maintaining muscle thickness to protect against immobilization-induced muscle atrophy^21^. Luo et al.^17^ suggested that even slight diaphragm muscle contractions during MV can protect against VIDD. The negative results in the present study may have been due to an insufficient number of samples.

There were several limitations of the present study. First, this was a pilot study with a small sample size. Furthermore, the results of this study were based on microarray gene analysis and not on observations of changes in proteins, muscle contractility, or muscle fibers. Further studies are needed to confirm whether ES reduces inflammatory cytokine levels, improves mitochondrial respiration, and counteracts skeletal muscle degradation. The mechanisms of VIDD in clinical settings are diverse and involve a complex interplay of various factors. Underlying conditions requiring MV, hyperglycemia, medication regimens, metabolic stress, coexisting illnesses, and imbalances in protein metabolism all have the potential to contribute to VIDD. Therefore, importantly, a broader understanding of these factors is needed to ensure the safer and more appropriate use of ES as a therapeutic approach for these patients in the future.

In conclusion, 12 h of MV shifts gene expression in the diaphragm muscle toward muscle degradation, and ES may counteract this shift by suppressing catabolic processes and improving mitochondrial respiration.

## Data Availability

All data obtained from the protocols and genetic analyses developed in this study are available from the corresponding author upon reasonable request.
